# Detection of Microsatellite Instability in Colorectal Cancer Patients With a Plasma-Based Real-Time PCR Analysis

**DOI:** 10.3389/fphar.2021.758830

**Published:** 2021-12-08

**Authors:** Namjoo Kim, Sung Min Kim, Beom Jae Lee, Byung il Choi, Hee Sook Yoon, Sang Hee Kang, Seung Han Kim, Moon Kyung Joo, Jong-Jae Park, Chungyeul Kim

**Affiliations:** ^1^ Department of Gastroenterology, Korea University Guro Hospital, Seoul, South Korea; ^2^ Department of Surgery, Korea University Guro Hospital, Seoul, South Korea; ^3^ Department of Pathology, College of Medicine, Korea University, Seoul, South Korea

**Keywords:** microsatellite instability, real-time PCR, colorectal cancer, mismatch repair, liquid biopsy

## Abstract

A microsatellite instability (MSI) test is crucial for screening for HNPCC (Hereditary nonpolyposis colorectal cancer; Lynch syndrome) and optimization of colorectal cancer (CRC) treatment. Mismatch repair (MMR) deficiency is a predictor for good response of immune checkpoint inhibitors in various malignancies. In this study, we evaluated the results of a newly developed plasma-based real-time PCR kit for the detection of MSI in CRC patients. We assessed a peptide nucleotide acid (PNA) probe-mediated real-time PCR test (U-TOP MSI Detection Kit Plus) that determines MSI status by using amplicon melting analysis of five markers (NR21, NR24, NR27, BAT25, and BAT26) from plasma. Eighty-four CRC patients (46 dMMR and 38 pMMR) with colorectal cancer were analyzed. The concordance rate of MSI status assessment between the plasma kit and IHC was 63.0% in dMMR patients (29/46), but in the pMMR evaluation, a 100% (38/38) concordance rate was observed. In the evaluation of the performance of a custom tissue U-TOP MSI Detection Kit and plasma kit in 28 patients, sensitivity, specificity, PPV (positive predictive value) and NPV (negative predictive value) of plasma kit were 68.4, 100, 100, and 44.4%, respectively, with the tissue U-TOP MSI Detection Kit. Our results demonstrate the feasibility of a non-invasive and rapid plasma-based real-time PCR kit (U-TOP MSI Detection Kit Plus) for the detection of MSI in colorectal cancer.

## Introduction

Colorectal cancer (CRC) is one of the most common cancers worldwide, and although screening methods and treatments have been improved, high morbidity and mortality are still observed. Colorectal cancer affects more than one million people each year, and disease-specific mortality is reported to be nearly 33% in developed countries (Arnold et al., 2017; Rawla et al., 2019; Wong et al., 2019; Bray et al., 2020; Siegel et al., 2020).

TNM stage remains a major determinant of patient prognosis after surgical resection of advanced CRC and informs treatment decisions. However, there is significant step-independent variability in clinical outcomes due to molecular heterogeneity. This variability highlights the need for robust prognostic and predictive biomarkers to guide therapeutic decision making, including the use of adjuvant chemotherapy (Thibodeau et al., 1993; Boland et al., 1998; Nguyen et al., 2020). Most CRCs occur through the chromosomal instability pathway, but approximately 12–15% are caused by DNA mismatch repair, characterized in tumors by microsatellite instability (MSI) (Boland et al., 1998; Nguyen et al., 2020).

Tumors with deficient mismatch repair (dMMR)/MSI can result from germline mutations in the MMR genes (MLH1, MSH2, MSH6, PMS2), i.e., Lynch syndrome, or more often from epigenetic inactivation of the MMR genes. CRC with dMMR has characteristic phenotype features including predominant proximal location, mostly poor differentiation, mucinous and lymphocyte deposition in the tumor and surrounding areas (Crohn-like cancer) (Thibodeau et al., 1993; Miyakura et al., 2001; Søreide et al., 2009). MSI-H/dMMR CRCs are associated with lack of responsiveness to conventional chemotherapy and there was a lack of benefit from adjuvant five FU-based chemotherapy in patients with stage II or III MSI-H/dMMR CRC (Hutchins et al., 2010; Kaya et al., 2017; Sargent et al., 2010; Paulose et al., 2019). Therefore, it is recommended to perform MSI testing as a routine examination to predict prognosis and therapeutic response to chemotherapy in the patients with CRC.

Evaluation of MMR status is important for predicting treatment response to immune checkpoint inhibitors (Le et al., 2015). The Food and Drug Administration (FDA) approved pembrolizumab, immunotherapy targeting the programmed cell death protein one receptor of lymphocytes, as a first-line drug for inoperable or metastatic MSI-H/dMMR colorectal cancer. Since MSI-H/dMMR is a predictor of a good response to immune check point inhibitors in several solid tumors, there is an increasing demand for methods that can evaluate MSI status with high flexibility and reliability (Overman et al., 2017; Sahin et al., 2019).

MMR status is clinically evaluated through immunohistochemistry of, MLH1, MSH2, MSH6, and PMS2 in tumor tissue, or through PCR-based analysis for detection of MSI from paraffin block tumor tissue. IHC-based MMR evaluation is performed to assess the loss of MMR proteins. During the interpretation process, the assessment of MMR status can be influenced by the pathologist’s subjective interpretation and technical factors. This method requires an invasive procedure to obtain tissue and is limited in the case of patients who cannot provide tissue (Joosse and Pantel, 2016; Tieng et al., 2021). Detection of MSI by PCR through fragment analysis, such as endoscopic biopsy, may not be appropriate because samples from both tumor and normal tissues are required. In addition, analysis by PCR requires an experienced team, requires analysis equipment, and has low sensitivity to samples with a low percentage of cancer cells. Research to find diagnostic markers and prognostic factors of tumors through liquid biopsy is ongoing. In colorectal cancer, research has been conducted to utilize it for blood-based diagnosis and screening, and its usefulness has been reported in some cases (You et al., 2010; Ferreira et al., 2016; Habli et al., 2020; Tieng et al., 2021). Therefore, the MSI molecular test has been suggested for blood samples and many studies are currently being conducted on its efficacy, but further research is still needed on its diagnostic value and clinical utility (Mokarram et al., 2014; Silveira et al., 2020). This study aims to analyze the usefulness of the newly developed real-time PCR-based MSI test from plasma in colorectal cancer patients who have undergone clinical MSI evaluation.

## Methods

### Patients and Samples

From December 2005 to July 2012, 84 patients diagnosed with colorectal cancer were included. The patients were analyzed for age, gender, and histopathological findings, and histopathological tissue and plasma were obtained from the Korea Human Resources Bank (Pusan National University Hospital, Gyeongsang National University Hospital, Inje University Hospital, and Korea University Guro Hospital). Written consent was waived because our study was a retrospective observational cohort and included non-therapeutic interventions. This study was approved by the Institutional Review Board at Korea University Guro Hospital.

### U-TOP MSI Detection Kit Plus

We used a peptide nucleotide acid (PNA) probe-mediated real-time PCR-based MSI test, U-TOP MSI Detection Kit Plus (Seasun Biomaterials, Daejeon, Korea), to detect MSI status. In PNA methods, a wild-type PNA probe is perfectly hybridized to monomorphic mononucleotide repeat markers and inhibits PCR amplification of the wild-type allele whereas mutant DNA with deletions is selectively enriched. This PCR method with PNA increases sensitivity. U-TOP MSI Detection Kit Plus can detect MSI status by using amplicon melting analysis of five quasi-monomorphic mononucleotide repeat markers (NR21, NR24, NR27, BAT25, and BAT26) and an internal control.

### DNA Extraction From Plasma and Formalin Fixed Paraffin Embedded Tissue

We extracted cell-free DNA and FFPE DNA from plasma and naïve slides of CRCs patients using the QIAamp Circulating Nucleic Acid Kit and the QIAamp DNA FFPE Tissue Kit (Qiagen, Valencia, CA, United States) individually. Quality and quantity of extracted FFPE DNA were evaluated by Nanodrop (ThermoFisher, MA, United States). The concentration of the extracted cfDNA was measured using an Agilent 2100 Bioanalyzer and an Agilent High Sensitivity DNA chip. The U-TOP MSI Detection Kit Plus includes primers and probes targeting ACTB as an internal control, allowing analysis of cfDNA concentration and quality.

### PNA Probe-Mediated Real-Time PCR-Based MSI Detection

To analyze the MSI status of CRCs, U-TOP MSI Detection Kit Plus based on PNA-mediated real-time PCR was used according to the manufacturer’s instruction. A 20-μl mixture composed of template DNA 3 μl, 2x qPCR Premix 10 μl and dual-labeled PNA probes for NR21, NR24, BAT26 and internal control (MSI marker A) 7 μl. Another 20-μl mixture was prepared using dual-labeled PNA probes for BAT25, NR27 and internal control (MSI marker B) with the same recipe as MSI marker A described above. The 2x qPCR Premix contained dNTPs, DNA Taq polymerase and Uracil DNA Glycosylase (UDG). MSI markers A and B contained primers and PNA probes to amplify five quasi markers and an internal control. These PNA probes were fluorescently labeled with FAM, HEX, Texas Red and Cy5. PCR reactions were performed for each normal and cancer sample using a CFX96 PCR machine (Bio-Rad, Hercules, CA, United States). PCR reaction steps consisted of amplification and subsequent melting point analysis. The amplification conditions were 50°C for 5 min, 95°C for 10 min, and 60 cycles of 95°C for 30 s, 65°C for 20 s, 54°C for 30 s, and 57°C for 30 s. The initial incubation at 50°C for 5 min was required to activate Uracil DNA Glycosylase and prevent possible carryover contamination. In 60 cycles of PCR, four different temperatures steps and respective optimal times were required. The main purpose of using these conditions was to increase sensitivity by inhibiting the wild allele and enriching the mutant allele with deletion in target MSI markers. In detail, 95°C for 30 s was for denaturation of template DNAs, 65°C for 20 s was for binding of PNA probes to their target MSI markers with deletion, 54°C for 30 s was for annealing of primers, and 57°C for 30 s was for elongation. The melting point analysis conditions were 3 min at 95°C for denaturation and touchdown PCR (90–45°C, decreasing 1°C per cycle). Fluorescence was measured for 10 s at each touchdown PCR cycle. Melting peaks obtained after amplification were analyzed to detect alterations in the five MSI marker genes. The sample was considered to be unstable in a MSI marker gene when the melting temperature was below the criteria. In the case of no melting peaks or temperature in the range of criteria, it considered as stable in a MSI marker gene by checking the internal controls amplification or melting peak. DNA samples from CRC patients were classified as MSI-H if instability was found in two or more of the five markers, MSI-L if instability was found in one marker, and MSS if no instability was present.

### Data Analysis

All statistical analyses were performed using the Statistical Package for the Social Sciences (SPSS) for Windows (version 20.0; IMB Corp., Armonk, NY, United States). The tissue plasma U-TOP MSI Detection Kit Plus assay was considered as the gold standard. Sensitivity and specificity of plasma U-TOP MSI Detection Kit Plus were calculated.

## Result

### Baseline Characteristics of Enrolled Patients

Plasma was obtained from a total of 84 patients with CRC with clinically evaluated MMR status. All patients’ MMR status was assessed by IHC. The mean age of all patients was 67.5±11.7 and there were 42 right colon cases (50%). There were 25 (29.7%) patients with left colon tumors and 17 (20.3%) patients with rectum tumors. At the time of diagnosis, nine patients (18.6%) were stage I, 40 patients (48.8%) were stage II, 32 patients (20.9%) were stage III, and five patients (11.6%) were stage IV. The patient’s clinical data are summarized in Table 1.

**TABLE 1:** Basal clinicopathological characteristics of enrolled patients.

	All patients (*n* = 84)
Sex, *n*	
Male	50 (59.5%)
Female	34 (40.5%)
Age, y	
Mean (SD)	67.5±11.7
Clinical stage, *n*	
I	9 (18.6%)
II	40 (48.8%)
III	32 (20.9%)
IV	3 (11.6%)
Location of tumor, *n*	
Rt. colon	42 (50.0%)
Lt. colon	25 (29.7%)
Rectum	17 (20.3%)
MSI status	
MSS	37 (11.6%)
MSI-L	1 (25.6%)
MSI-H	46 (62.8%)

### Diagnostic Sensitivity and Specificity of U-TOP MSI Detection PCR

**FIGURE 1:**
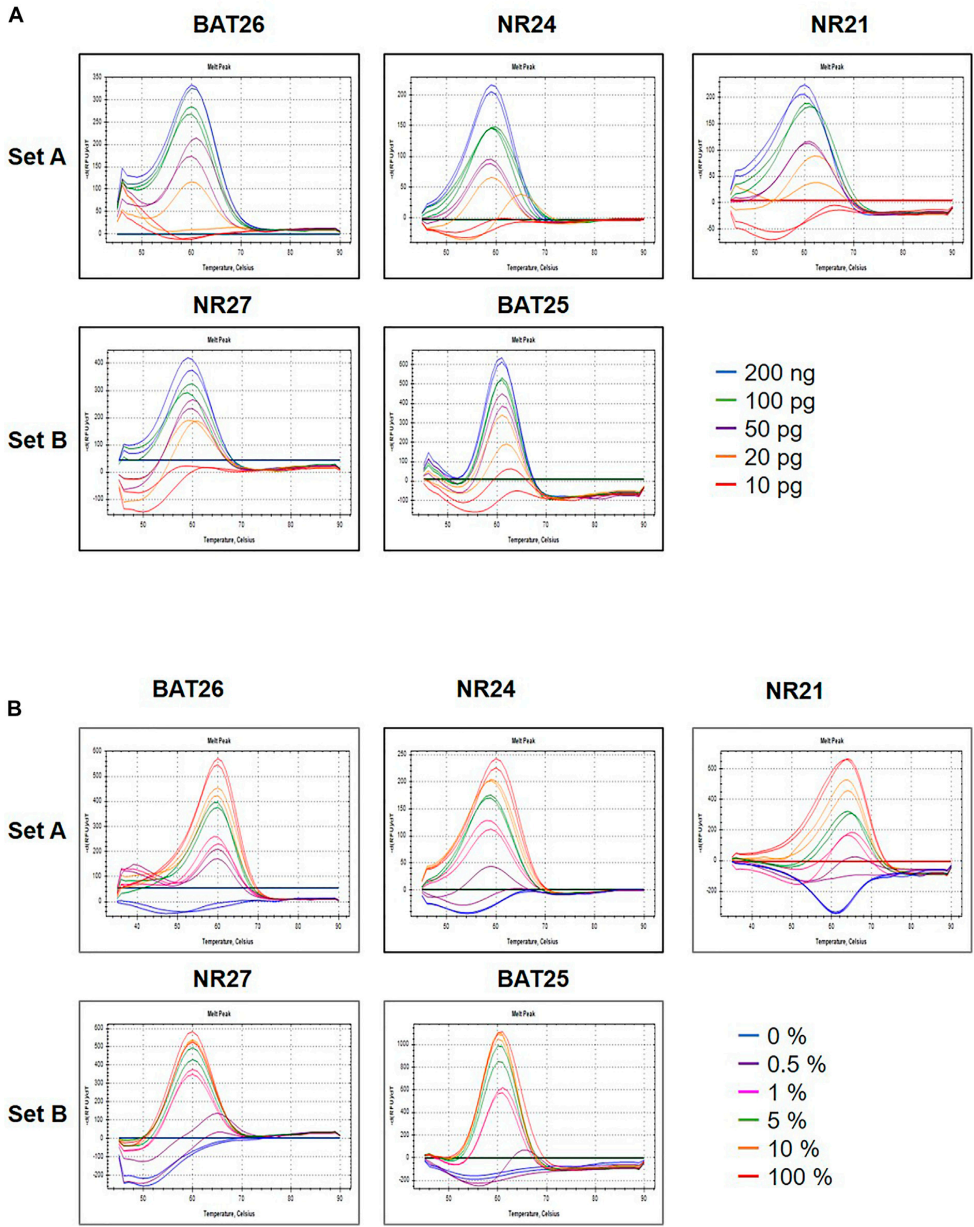
Maximum sensitivity evaluation of PNA method. The maximum sensitivity of PNA methods was evaluated using mixed samples of genomic DNA sample obtained from HeLa (MSS) and SNU-1634 (MSI-H) cells. **(A)** A PNA method was capable of detecting alteration in all five MSI marker genes in sample containing down to 50 pg concentration **(A)** and **(B)** down to 1% MSI-H variant.

To determine the minimum concentration detection limit of the U-TOP MSI Detection Kit Plus, a clinical plasma sample previously identified as d MMR was used, and the sample was diluted to 200, 100, 50, 20, or 10 pg. Specific melting temperature values were confirmed up to 20 pg in some markers (BAT25, NR24), TM was confirmed up to 50 pg in BAT26 and NR24, and the minimum detection limit of the U-TOP MSI Detection Kit Plus was set to 50 pg (Figure 1A). To assess the MSI sensitivity of the U-TOP MSI Detection Kit Plus, MSI-H cell line DNA (SNU-1634) and MSS cell line DNA (Hela Genomic DNA, NEB) were used. Each DNA was mimicked by MSI percent (0, 0.5, 1, 5, 10, and 100%), and 50 pg of DNA was used for the test. The results showed BAT25 detectable up to 0.5% of MSI, but in other markers, specific TM values of each marker were confirmed up to 1% of MSI, and TM values were not confirmed in percentages below. Thus, the MSI detection sensitivity of the U-TOP MSI Detection Kit Plus was set to 1% (Figure 1B).

### Determination of MSI Status by the Plasma and Tissue U-TOP MSI Detection PCR

**FIGURE 2:**
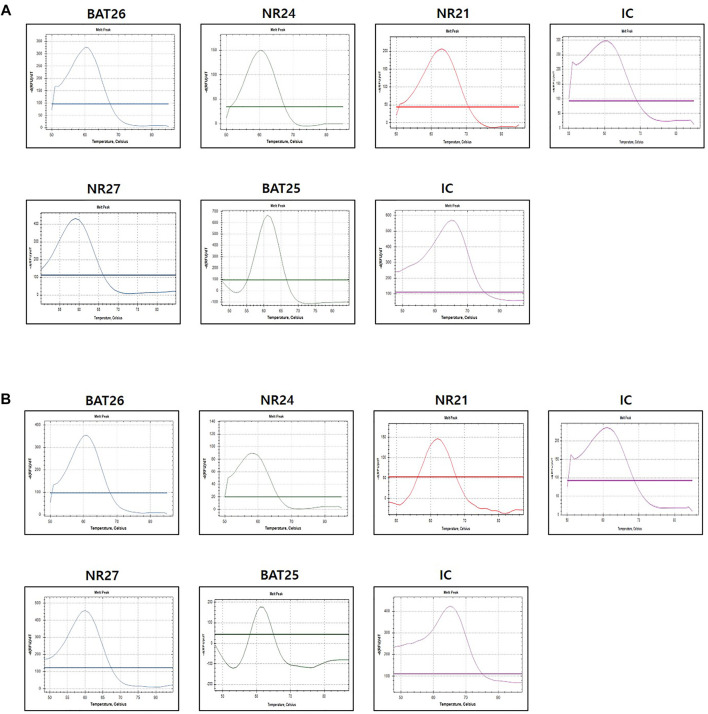
Representative MSI test using PNA-mediated melting point analysis (case 20) **(A)** results from FFPE sample **(B)** result from plasma sample. Alterations were detected in all five markers in both FFPF and plasma sample.

**FIGURE 3:**
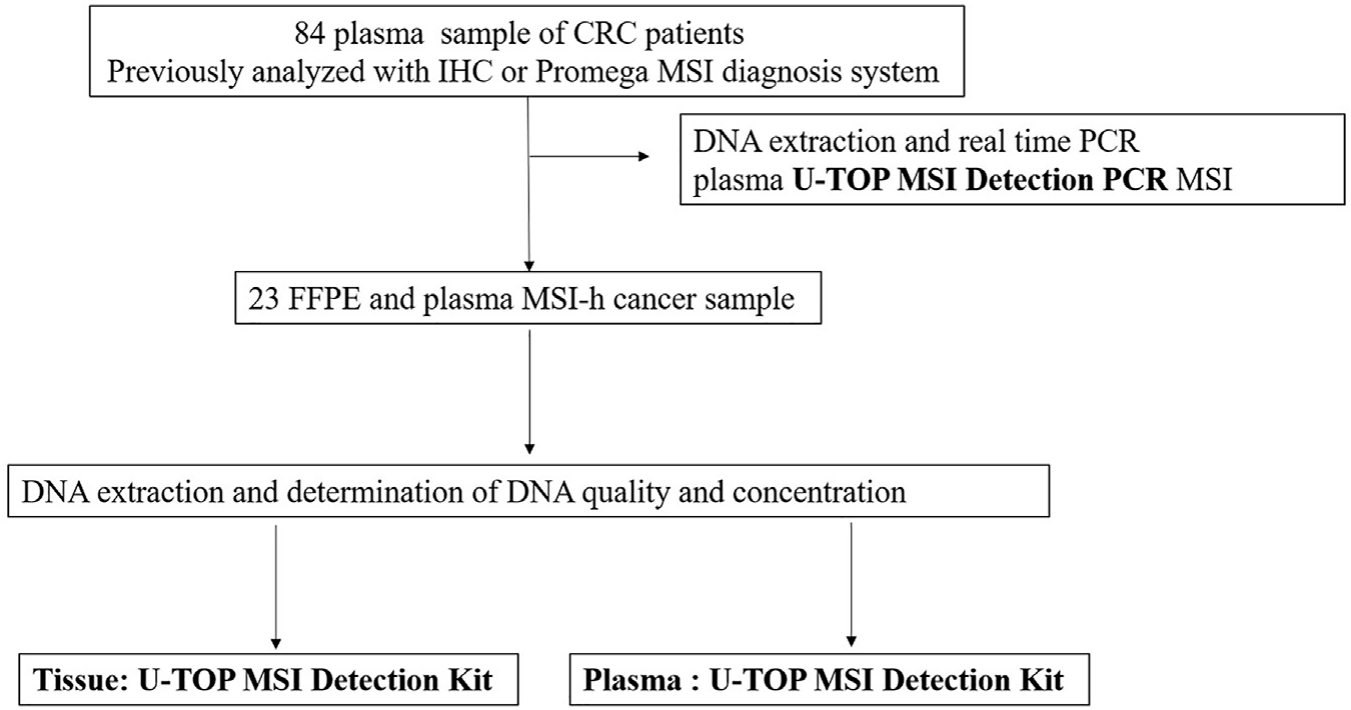
Flow chart of the study.

The evaluation of MSI was performed by the PNA method, which is a real-time PCR-based method. Samples with observed mutations in two or more MSI marker were determined as MSI-H and samples with or without alterations in a single MSI marker were determined as MSI-L or MSS, respectively (Figures 2A,B). PCR analysis was performed on the plasma of 84 patients using the newly developed U-TOP MSI Detection Kit (Figure 3). Of 38 patients diagnosed with pMMR, 37 had MSS and one had MSI-L at IHC. In 37 patients with MSS, 28 patients were identified as MSS and nine patients as MSI-L by the plasma U-TOP kit, and one patient with MSI-L was matched by plasma U-TOP kit. In dMMR patients, the concordance rate of MSI status evaluation between the plasma kit and IHC was 63.0% (29/46), but in the pMMR evaluation, a concordance rate of 100% (38/38) was observed.

### Concordance Between the Results of Tissue and Plasma U-TOP MSI Detection PCR

For 23 patients, the agreement between the tissue U-TOP MSI kit and the developed plasma kit was compared and analyzed (Table 2). Thirteen of 19 patients diagnosed with MSI-H by the Tissue U-TOP MSI Kit were determined to be MSI-H by plasma PCR, and a concordance rate of 68.4% was observed (Table 3). Among four patients diagnosed with pMMR (three MSS, one MSI-L) by Tissue U-TOP MSI Kit, MSI-L was determined as MSS by the plasma kit (case 4), but the evaluation of tissue and plasma in the pMMR diagnosis was consistent with the both test kits. Regarding the concordance rates for each microsatellite locus, NR24 and BAT25 concordance rates were 77.2 and 72.7%, respectively, showing the highest concordance rate among genes, and the lowest concordance rate was observed with NR21 (54.5%).

**TABLE 2:** Comparison of MSI status and concordance by Tissue: U-TOP MSI Detection Kit and plasma U-TOP MSI Detection PCR.

Case	Gender	Age	Location	Size (cm)	Stage			Pathology
					T	N	M	
Case 1	M	74	Left	8	3	0	0	Moderately differentiated
Case 2	F	64	Right	11	1	0	0	Moderately differentiated
Case 3	F	74	Rectum	2	3	0	0	Moderately differentiated
Case 4	M	63	Left	2.7	3	1A	1	Poorly differentiated
Case 5	M	81	Right	8.1	3	1B	0	Moderately differentiated
Case 6	F	64	Left	7	3	0	0	Well differentiated
Case 7	F	73	Right	4	2	0	0	Poorly differentiated
Case 8	F	57	Right	8.0	3	2b	0	Poorly differentiated
Case 9	M	60	Right	3	3	0	0	Moderately differentiated
Case 10	F	63	Right	5.5	2	0	0	Mucinous adenocarcinoma
Case 11	M	82	Right	7	2	1B	0	Moderately differentiated
Case 12	M	79	Right	3.3	2	0	0	Moderately differentiated
Case 13	M	70	Left	6.7	3	2B	0	Mucinous adenocarcinoma
Case 14	F	43	Right	9	3	1B	0	Moderately differentiated
Case 15	M	75	Right	7	3	1B	0	Mucinous adenocarcinoma
Case 16	F	77	Right	8	2	2b	0	Moderately differentiated
Case 17	M	80	Right	9.5	3	2B	0	Moderately differentiated
Case 18	F	68	Right	7	0	0	1	Moderately differentiated
Case 19	F	55	Rectum	8	2	0	0	Moderately differentiated
Case 20	M	99	Left	9	3	0	0	Poorly differentiated
Case 21	M	62	Right	9.5	2	0	0	Well differentiated
Case 22	M	86	Right	2	4	0	1	Moderately differentiated
Case 23	F	75	Rectum	2	3	0	0	Moderately differentiated

Case	Tissue: U-TOP MSI Detection Kit						Plasma: U-TOP MSI Detection Kit v2						cfDNA Con. (pg/μl)	Concordance
	BAT	NR	NR	NR	BAT	MSI	BAT	NR	NR	NR	BAT	MSI		
	26	24	21	27	25		26	24	21	27	25			
Case 1	+	+	+	+	+	MSI-H	+	−	+	−	+	MSI-H	182	O
Case 2	+	−	+	+	+	MSI-H	−	−	−	−	+	MSI-L	105	X
Case 3	−	+	+	−	+	MSI-H	+	+	+	−	+	MSI-H	201	O
Case 4	−	−	−	−	−	MSS	−	−	−	−	+	MSI-L	235	X
Case 5	−	+	+	+	+	MSI-H	+	+	+	+	+	MSI-H	193	O
Case 6	−	−	−	−	−	MSS	−	−	−	−	−	MSS	92	O
Case 7	+	−	−	−	−	MSI-L	+	−	−	−	−	MSI-L	134	O
Case 8	+	+	+	+	+	MSI-H	+	+	+	+	+	MSI-H	126	O
Case 9	−	−	−	−	−	MSS	−	−	−	−	−	MSS	105	O
Case 10	+	+	+	+	-	MSI-H	+	+	−	−	+	MSI-H	193	O
Case 11	+	+	+	+	+	MSI-H	−	+	+	+	+	MSI-H	211	O
Case 12	+	+	+	−	+	MSI-H	+	−	−	−	−	MSI-L	68	X
Case 13	+	+	+	+	+	MSI-H	+	+	+	−	+	MSI-H	168	O
Case 14	+	−	−	+	+	MSI-H	−	−	−	−	+	MSI-L	95	X
Case 15	+	+	+	+	+	MSI-H	−	−	−	−	-	MSS	71	X
Case 16	+	+	+	+	+	MSI-H	+	+	−	−	+	MSI-H	103	O
Case 17	+	+	+	+	+	MSI-H	+	+	+	−	+	MSI-H	126	O
Case 18	+	+	+	+	+	MSI-H	−	+	−	+	+	MSI-H	122	O
Case 19	+	+	+	+	+	MSI-H	−	+	+	+	−	MSI-H	145	O
Case 20	+	+	+	+	+	MSI-H	+	+	+	+	+	MSI-H	85	O
Case 21	−	+	−	+	+	MSI-H	−	−	−	+	−	MSI-L	81	X
Case 22	+	+	−	−	+	MSI-H	+	+	−	+	+	MSI-H	147	O
Case 23	+	+	−	−	+	MSI-H	−	−	−	−	+	MSI-L	108	X

**TABLE 3:** Concordance rate between plasma U-TOP MSI Detection PCR and tissue U-TOP MSI Detection PCR.

Plasma U-TOP MSI Detection PCR		Tissue: U-TOP MSI Detection Kit		Sum
		MSI	MSS	
U-TOP	MSI	13	0	13
	MSS	6	4	10
Sum		19	4	23

### Concordance Rate of Plasma Kit According to Clinical Features

When the tumor was classified by stage, the concordance rate of the plasma kit was 72.7% for stage III and IV patients and 66.6% for early stage patients. In classification according to tumor location, a concordance rate of 75% was observed for tumors located in the left colon, and a concordance rate of 66.6% for right colon cancer. In the agreement rate according to the size of the tumor, no difference was observed in the agreement rate according to the size of the intestinal tract (6.4 ± 2.8 vs. 6.35 ± 2.8 cm, *p* = 0.177).

## Discussion

Determination of MSI/MMR status is now standard of care for all CRC patients (Umar et al., 2004). In 2017, the FDA approved the use of pembrolizumab for the patient with unresectable or metastatic, MSI-high CRC that did not respond to fluoropyrimidines, such as 5-FU, oxaliplatin, or irinotecan (Sahin et al., 2019). In 2020, the FDA approved pembrolizumab in the frontline setting for patients with metastatic MSI-H/dMMR CRC based upon the findings from the Keynote-177 findings (André et al., 2020).

In the evaluation of MMR, IHC staining for MLH1, PMS2, MSH2, and MSH6 proteins and a PCR-based MSI detection system obtained from tissue are being applied in the clinical field for the determination of MMR status (Jang et al., 2018; Gilson et al., 2020). The U-TOP kit is a high-sensitivity kit adopting real-time PCR technology designed to detect five MSI markers in human plasma cell-free DNA (cfDNA) samples. In the present study, we evaluated the consistency of MMR evaluation in plasma cfDNA samples from advanced colorectal cancer by comparing the plasma U-TOP kit, tissue PCR, and clinically obtained IHC results. Our study is the first to evaluate the performance of the plasma U-TOP kit in the plasma MSI molecular test. MSI was evaluated using 84 blood samples from colorectal cancer patients, and comparative analysis was performed with the PCR results obtained from 23 FFPE tissues. The U-TOP kit showed a sensitivity of 69% and a specificity of 100% based on the results of tissue PCR. cfDNA can be measured as it enters the bloodstream during apoptosis or necrosis in normal and tumor cells. The length of the cfDNA strand varies depending on the tumor characteristics. Compared to normal cells, cfDNA is released at a much larger length by necrosis in tumor cells (Jahr et al., 2001; Oh and Joo, 2020). By qualitative real-time PCR, cfDNA showed a sensitivity of 73.08% and a specificity of 97.27% for colorectal cancer diagnosis. CRC patients had 5-fold higher serum cfDNA concentration and 25–50-fold higher plasma cfDNA concentration compared to normal controls (Hao et al., 2014). A meta-analysis of circulating cfDNA for diagnosis of CRC showed a sensitivity of 73.5% and a specificity of 91.8%, indicating acceptable specificity for diagnosis of CRC (Wang et al., 2018).

According to previous studies, the average cfDNA proportion is 4.5% (Rossi et al., 2018), and the ctDNA level can be affected by various factors including tumor burden and treatment process (Schwaederle et al., 2016). The correspondence rate between MSI-H tissue and cfDNA activities was reported to be 87 and 99.5% (Willis et al., 2019). Factors for discrepancy include tumor heterogeneity, differential shedding by the primary versus metastatic lesions, temporal discordance of tissue and plasma collection, and low tumor shedding by some tumors (Schwaederle et al., 2016). The present study showed concordance rate of about 70%, lower than in previous studies. The concentration of cfDNA in the sample used in this study was higher than the determined cut-off value and was appropriate, and the samples were obtained immediately before surgery to rule out an effect on the concentration of cfDNA by treatment. One of the presumed reasons for the relative low concordance rate is its long storage period, because this study analyzed samples deposited in human tissue banks. Also, differential individual tumor characteristics such as degree of necrosis and any factors that decrease tumor shedding are possible sources of variability. Our real-time-based PCR platform has limited ability to capture cfDNA from samples from CRC patients. However, if the patient’s condition makes it difficult to obtain a biopsy for the lesion or if other procedures are not required, a liquid biopsy is advantageous and may be an alternative method to assess MSI status. Further research on the factors that affect detection of plasma MSI status is needed.

The advantages of liquid biopsy, such as blood, are that it can overcome the inherent limitations of the assay from other tissues such as invasiveness, time-consuming, and the heterogenous nature of colorectal cancer, and can be used for predicting treatment response and screening tests. CRC with MSI-His known to be more frequent in stage II compared to stage III and relatively rare, accounting for about 4% of metastatic tumors (Roth et al., 2010; Kawakami et al., 2015). This blood-based MSI test can be helpful in the evaluation of MSI in metastatic CRC patients who cannot undergo tissue biopsy. In addition, it can be used as a screening tool in the health check-up process for early detection of Lynch syndrome, and can be used as an MSI test in a relative with Lynch syndrome. It might also be used as an MSI test in patients with other unresectable tumors such as metastatic endometrial and pancreatic cancer. Studies on the effectiveness of MSI diagnosis using liquid biopsy have been actively reported in MSI tests (Tieng et al., 2021). The study of NGS-based liquid biopsy is still limited to the academic field because it is expensive, takes a long time to analyze, and has limitations in interpreting data used in bioinformatics (Niu et al., 2014; Liu et al., 2019). The detection rate of MSI in multiplex and real-time PCR-based liquid biopsies did not show 100% sensitivity, including in this study, which is due to the fact that cfDNA is a mixture of different DNAs with a trace amount of cancer-derived DNA, making it practically impossible to detect in plasma of all cancer patients. MSI detection through real-time PCR is reported to be inferior to ddPCR (droplet digital PCR). ddPCR is 1,000 times more sensitive than conventional real-time PCR, so it is easy to analyze target genes in a tumor-like mixture or heterogenous state. However, economic feasibility, compatibility with existing PCR equipment, and the need for a specialized laboratory to interpret data are problematic, so there are still some limitations in applying it to the clinical field.

This study had several limitations. First as mentioned above, the MSI-H status was not captured by our system in about 30% of patients with CRC. A prospective study with large scale will be needed to identify the causes that may affect the MSI determination result of our PCR-based kit including the characteristics of individual CRC that are not captured by this PCR kit. Second, we did not test solid tumors other than colorectal cancer, and third, comparative analyses with other liquid biopsy methods such as NGS and ddPCR were not performed.

The plasma U-TOP MSI Detection Kit Plus kit used in this study has a sensitivity of about 70% and a positive predictive rate of 100%, showing superior results to the existing real-time-based PCR method. It has a short analysis time of about 4 h, economic advantages of using existing equipment, and is a clinically applicable test method. The positive predictive rate is also excellent, so it is judged that it can be applied to screening tests considering the advantage that it can be analyzed quickly and easily if applied complementary to existing tests such as IHC analysis.

In conclusion, a new PNA-based real-time PCR kit allowed the detection of MSI status in the plasma of CRC patients. A plasma-based MSI test can be an effective method to confirm MSI status in patients with suspected Lynch syndrome and their immediate family, colorectal tumors resulting from the serrated pathway (Crockett and Nagtegaal, 2019; Kahi, 2019). With additional research, it could be an alternative diagnostic method to check MSI status in patients with difficult tissue collection or limited surgical treatment in other solid cancer patients.

## Data Availability

The original contributions presented in the study are included in the article/Supplementary Material, further inquiries can be directed to the corresponding author.

## Abbreviation

cfDNA, cell-free DNA; CRC, colorectal cancer; ddPCR, droplet digital PCR; dMMR, deficient mismatch repair; FFPE, formalin fixed paraffin embedded; HNPCC, hereditary nonpolyposis colorectal cancer; MMR, mismatch repair; MSI, microsatellite instability; PNA, peptide nucleotide acid; pMMR, proficient mismatch repair.
